# Supplementary material for: Dynamic control of hybrid grafted perfect vector vortex beams
Source: Nat Commun. 2023 Jul 3;14:3915. doi: 10.1038/s41467-023-39599-8 (PMC10318044; doi:10.1038/s41467-023-39599-8)
Supplement: Supplementary file 1 — Supplementary Information [file 41467_2023_39599_MOESM1_ESM.pdf]

Supplementary Information: Dynamic Control of Hybrid Grafted Perfect Vector Vortex Beams

Hammad Ahmed [1], Muhammad Afnan Ansari [1], Yan Li [1,2], Thomas Zentgraf[3], Muhammad Qasim Mehmood [4] and Xianzhong Chen [1,]*

[1]Institute of Photonics and Quantum Sciences, School of Engineering and Physical Sciences, Heriot-Watt University, Edinburgh EH14 4AS, UK

[2]School of Materials, Zhengzhou University of Aeronautics, Zhengzhou 450015, China

[3]Paderborn University, Department of Physics, Warburger Str. 100, 33098 Paderborn, Germany

[4]MicroNano Lab, Electrical Engineering Department, Information Technology University (ITU) of the Punjab, Ferozepur Road, Lahore, 54600, Pakistan

*E-mail: x.chen@hw.ac.uk

Supplementary Section 1: Mathematical Analysis of GPVVBs

Perfect optical vortex (POV) beams and perfect vector beams (PVBs) are two types of structured beams. POVs have helical phase fronts with constant ring radius independent of TCs, while PVBs have spatially inhomogeneous polarization distributions. The scalar POVs can be mathematically expressed as [1, 2]

$$E = \exp\left[-\frac{(\rho-\rho_o)^2}{\Delta\rho^2}\right] e^{il\psi}, (S1)$$

here ρ_o is the ring radius, $\Delta\rho$ is the width of the ring, l is the topological charge and ψ is the azimuth angle. Whereas PVBs can be described as the superposition of two orthogonal circularly polarized scalar POVs with conjugated TCs[3]

$$\boldsymbol{E_{PVB}} = E_R e^{i(\varphi_o+\alpha)}|\boldsymbol{R}_m\rangle + E_L e^{-i(\varphi_o+\alpha)}|\boldsymbol{L}_{-m}\rangle, (S2)$$

where

$$|\boldsymbol{R}_m\rangle = \exp\left[-\frac{(\rho-\rho_o)^2}{\Delta\rho^2}\right] e^{im\psi}\begin{bmatrix}1\\-i\end{bmatrix},$$

and

$$|\boldsymbol{L}_m\rangle = \exp\left[-\frac{(\rho-\rho_o)^2}{\Delta\rho^2}\right] e^{-im\psi}\begin{bmatrix}1\\i\end{bmatrix}.$$

Where m is the polarization order, φ_o is the initial phase and α is the angle between the transmission axis of the input linear polarizer (LP) and the x-axis. $|\boldsymbol{R}_m\rangle$ and $|\boldsymbol{L}_m\rangle$ are the right- and left-handed components. E_R and E_L are the RCP and LCP amplitudes, respectively. Jones calculus is usually employed to investigate the transmission process and polarization distribution. The Jones vector for PVBs can be written as[4]

$$\boldsymbol{E} = \exp\left[-\frac{(\rho-\rho_o)^2}{\Delta\rho^2}\right]\begin{bmatrix}\cos(m\psi+\varphi_o+\alpha)\\ \sin(m\psi+\varphi_o+\alpha)\end{bmatrix}. (S3)$$

The amplitudes are ignored for simplicity. The same concept can be extended to PVVBs, which are the superposition of two orthogonal circularly polarized scalar OVs with unconjugated TCs. PVVBs can be expressed as[5]

$$\boldsymbol{E_{PVVB}} = E_R e^{i(\varphi_{o}+\alpha)}|\boldsymbol{R}_{l_a}\rangle + E_L e^{-i(\varphi_{o}+\alpha)}|\boldsymbol{L}_{l_b}\rangle, (S4)$$

where

$$|\boldsymbol{R}_m\rangle = \exp\left[-\frac{(\rho-\rho_o)^2}{\Delta\rho^2}\right]e^{il_a\psi}\begin{bmatrix}1\\ -i\end{bmatrix},$$

and

$$|\boldsymbol{L}_m\rangle = \exp\left[-\frac{(\rho-\rho_o)^2}{\Delta\rho^2}\right]e^{-il_b\psi}\begin{bmatrix}1\\ i\end{bmatrix}.$$

l_a and l_b are TCs for RCP and LCP OVs, respectively. Here, the polarization distribution and phase distribution can be obtained by polarization order $m = \frac{(l_a-l_b)}{2}$ and Pancharatnam charge $p = \frac{(l_a+l_b)}{2}$, respectively. Based on m and p, the Jones vector for PVVBs can be written as

$$\boldsymbol{E} = \exp\left[-\frac{(\rho-\rho_o)^2}{\Delta\rho^2}\right]e^{ip\psi}\begin{bmatrix}\cos(m\psi+\varphi_o+\alpha)\\ \sin(m\psi+\varphi_o+\alpha)\end{bmatrix}. (S5)$$

An arbitrary polarization order and phase distribution can be obtained by manipulating l_a and l_b. The polarization distributions can be revealed after light passes through an analyser, whose Jones matrix is given as[6]

$$\boldsymbol{J_{LP}} = \begin{bmatrix}\cos^2\alpha_A & cos\alpha_A sin\alpha_A\\ cos\alpha_A sin\alpha_A & sin^2\alpha_A\end{bmatrix}, (S6)$$

where, α_A is the angle between the transmission axis of the analyser and the x-axis. The resultant polarization state can be presented as

$$\boldsymbol{P} = \boldsymbol{J_{LP}}\boldsymbol{E_{PVVB}} = \exp\left[-\frac{(\rho-\rho_o)^2}{\Delta\rho^2}\right] e^{ip\psi}\begin{bmatrix}\cos(m\psi+\varphi_o+\alpha+\alpha_A)\cos(\alpha_A)\\ \cos(m\psi+\varphi_o+\alpha)\sin(\alpha_A)\end{bmatrix}. (S7)$$

Similarly, GPVVBs are formed by the combination of two orthogonal circularly polarized GPVBs

$$\boldsymbol{E_{GPVVB}} = E_R e^{i(\varphi_o+\alpha)}|\boldsymbol{R}_{GPVB_\mathrm{a}}\rangle + E_L e^{-i(\varphi_o+\alpha)}|\boldsymbol{L}_{GPVB_\mathrm{b}}\rangle, (S8)$$

where

$$|\boldsymbol{R}_{GPVB_\mathrm{a}}\rangle = \exp\left[-\frac{(\rho-\rho_o)^2}{\Delta\rho^2}\right] e^{i\varphi_{GVB_a}}\begin{bmatrix}1\\-i\end{bmatrix},$$

and

$$|\boldsymbol{L}_{GPVB_b}\rangle = \exp\left[-\frac{(\rho-\rho_o)^2}{\Delta\rho^2}\right] e^{-i\varphi_{GVB_b}}\begin{bmatrix}1\\i\end{bmatrix}$$

are the right circularly polarized (RCP) and left circularly polarized (LCP) GPVBs, respectively. ρ_o is the ring radius, $\Delta\rho$ is the width of the ring and φ_o is the initial phase. E_R and E_R are amplitudes of RCP and LCP components, respectively. φ_{GVB} can be given as

$$\varphi_{GVB} = \arg\left\{\exp\left[i\sum_{n=1}^{N} rect\left(\frac{N\psi}{2\pi}-\frac{N+1}{2}+\mathrm{a}n\right) l_n\psi\right]\right\}, (S9)$$

here, N and ψ are the total number of GVBs and the azimuth angle, respectively. The polarization distribution can be obtained by polarization order $m_n = \frac{(l_{an}-l_{bn})}{2}$ and the phase distribution is determined by the topological Pancharatnam charge $p_n = \frac{(l_{an}+l_{bn})}{2}$. The resultant polarization state for a particular sector can be written as

$$\boldsymbol{P_n} = \mathrm{e}^{ip_n\theta}\exp\left[-\frac{(\rho-\rho_o)^2}{\Delta\rho^2}\right]\begin{bmatrix}\cos(m_n\psi+\varphi_o+\alpha+\alpha_A)\cos(\alpha_A)\\ \cos(m_n\psi+\varphi_o+\alpha+\alpha_A)\sin(\alpha_A)\end{bmatrix}. (S10)$$

The above polarization state can be further modified with the help of HWP. The Jones matrix for HWP can be expressed as

$$J_{HWP} = \begin{bmatrix} cos2\beta & sin2\beta \\ sin2\beta & -cos2\beta \end{bmatrix}. (S11)$$

The jones vector for modified GVVB can be described as

$$E_{GPVVB_{new}} = J_{HWP}E_{GPVVB} = \exp\left[-\frac{(\rho-\rho_o)^2}{\Delta\rho^2}\right] e^{ip\psi} \begin{bmatrix} cos(2\beta - m_n\psi - \varphi_o - \alpha) \\ sin(2\beta - m_n\psi - \varphi_o - \alpha) \end{bmatrix}, (S12)$$

The above equation shows that the outgoing beam is still a GPVVB, but the polarization order is opposite to that of the resultant beam before passing through the HWP and the polarization direction is rotated by 2β.

The resultant polarization state is

$$P_{n_{new}} = J_{LP}E_{GPVVB_{new}}$$
$$= e^{ip_n\theta} \exp\left[-\frac{(\rho-\rho_o)^2}{\Delta\rho^2}\right] \begin{bmatrix} \cos(\alpha_A + 2\beta - m_n\psi - \varphi_o - \alpha)\cos(\alpha_A) \\ \cos(\alpha_A + 2\beta - m_n\psi - \varphi_o - \alpha)\sin(\alpha_A) \end{bmatrix}. (S13)$$

Supplementary Section 2: Polarization Manipulation

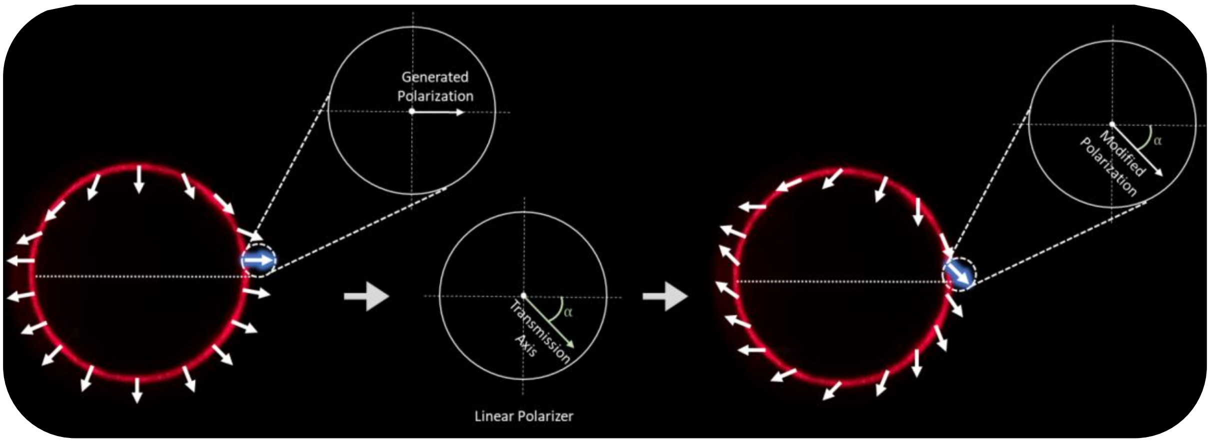

Fig. S1. Polarization control mechanism. Left: Intensity profile of GPVVB for the polarization order $m_1 = -1$ (upper sector) and $m_2 = +1$ (lower sector). The white arrows show the generated polarization profile. For the analysis purpose, a single polarization state (highlighted white arrow) is chosen as shown in the inset. The direction of a polarization state is the same as the x-axis. An incident polarization is varied with help of an LP. Middle: shows a schematic of LP. α is the angle between the transmission axis and x-axis. Right: Intensity profile of GPVVB with modified polarization state. By changing incident polarization by an amount α, the generated polarization profile for each sector is rotated by an angle α. The inset shows the modified version of a chosen polarization state.

Supplementary Section 3: Unit Cell Design

The proposed devices are realized based on plasmonic metasurfaces, which consist of gold (Au) nanorods with spatially varying orientations on a glass substrate as shown in **Fig. S2**. Such nanorods show strong light-matter interaction when they are resonantly excited at their localized surface plasmon polariton resonance. The length, width, and height of each nanorod are $\mathrm{L} = 0.2\ \mu\mathrm{m}$ long, $\mathrm{W} = 0.08\ \mu\mathrm{m}$, and $\mathrm{H} = 0.2\ \mu\mathrm{m}$, respectively. The periodicity (P) of each unit cell is $\mathrm{P} = 0.3\ \mu\mathrm{m}$ along both the x and y directions. The experimental and simulated efficiencies are illustrated in **Supplementary Section 4**. The simulated efficiency is calculated using the frequency domain solver of the Computer Simulation Technology (CST) Microwave Studio software. In the simulation, the permittivity of the gold nanorods is obtained by a Drude model with the plasma frequency $\omega_p = 1.37 \times 10^{16}$ rad/s, and the collision frequency $\gamma_c = 1.215 \times 10^{14}$ rad/s. The refractive index of the substrate is 1.46. The unit cell boundary is used along both the x and y directions, while the open boundary condition is used along the z direction. Although the cross-polarised part is low and the co-polarised part is high, the two parts are separated using the off-axis design as shown in the experimental setup in **Fig. S6.** Dielectric metasurfaces can be used to significantly improve the efficiency.

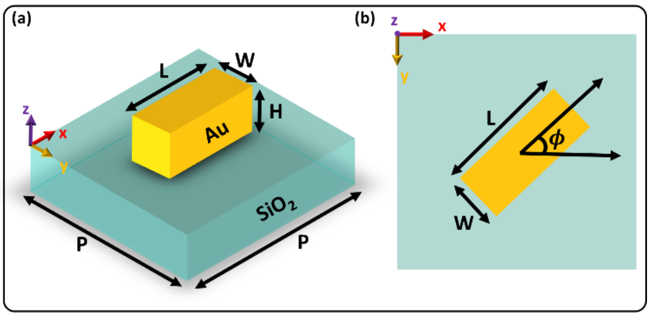

Fig. S2. Unit cell design. (a) Schematic of Au nanorods on a glass substrate. Length ($\mathrm{L} = 0.2\ \mu\mathrm{m}$), width ($\mathrm{W} = 0.08\ \mu\mathrm{m}$), height ($\mathrm{H} = 0.2\ \mu\mathrm{m}$), and periodicity ($\mathrm{P} = 0.3\ \mu\mathrm{m}$) are geometric parameters. (b) Top view of the unit cell with a nanorod rotated at an angle ϕ.

Supplementary Section 4: Metasurface Efficiencies

Diffraction Efficiency

The diffraction efficiency (η_D) can be defined as the ratio of the power of diffracted light (I_D) to the total power of transmitted light through the metasurface (I_T). Since the off-axis design

is used here, I_D is the same as the power of cross-polarised light. Therefore, I_T is the sum of the power of the co-polarised light (I_{co}) and that of the cross-polarised light (I_{cross}). Mathematically, η_D can be expressed as[7, 8, 9]

$$\eta_D = \frac{I_D}{I_T} \approx \frac{I_{cross}}{I_{co} + I_{cross}}. (S14)$$

The simulated and measured diffraction efficiencies for the proposed work are 34.3% and 13.9% at 633 nm, respectively. We also measure the diffraction efficiency at other wavelengths as illustrated in **Fig. S3.** As a proof-of-concept, we use plasmonic metasurfaces with a low efficiency, which can be dramatically improved using dielectric metasurfaces.

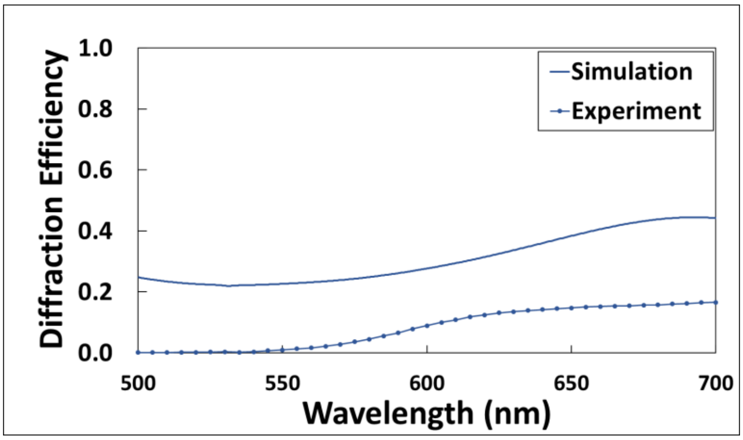

Fig. S3. Simulated and measured diffraction efficiency

Transmission Efficiency

The transmission efficiency is the ratio of total transmitted power through the metasurface (I_T) to the input power (I_{in}). I_T is the sum of the power of co-polarised light (I_{co}) and that of cross-polarised light (I_{cross}). The mathematical expression can be written as [7, 8, 9]:

$$\eta_T = \frac{I_T}{I_{in}} \approx \frac{I_{co} + I_{cross}}{I_{in}}. (S15)$$

The simulated and measured transmission efficiencies (**Fig. S4**) at 633 nm are 52.7% and 51%, respectively. The overall curves are relatively flat and uniform within the broadband ranging from 500 nm to 700 nm. It is worth noting here that the simulated and measured converted transmission efficiencies are 18% and 7%, respectively. For better understanding, we also plot I_{co} and I_{cross} separately in **Fig. S5**."

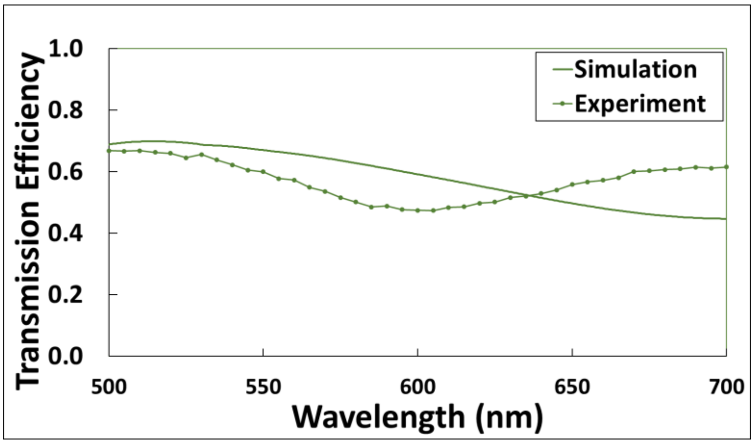

Fig. S4. Simulated and measured transmission efficiency.

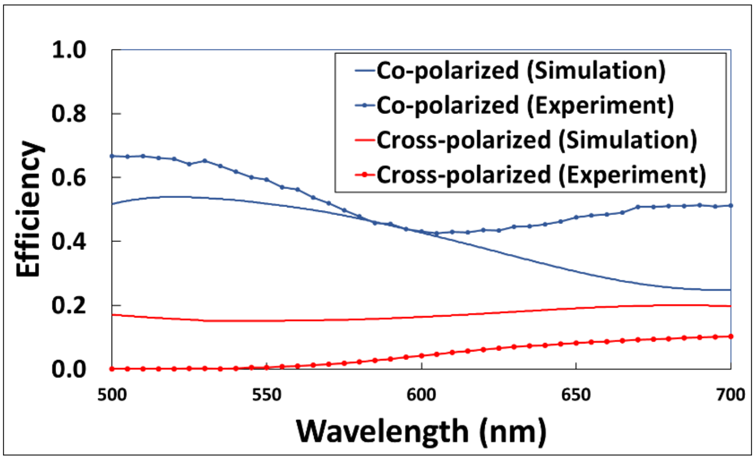

Fig. S5. Simulated and measured co-polarisation and cross-polarisation efficiency.

Supplementary Section 5: Optical Setup

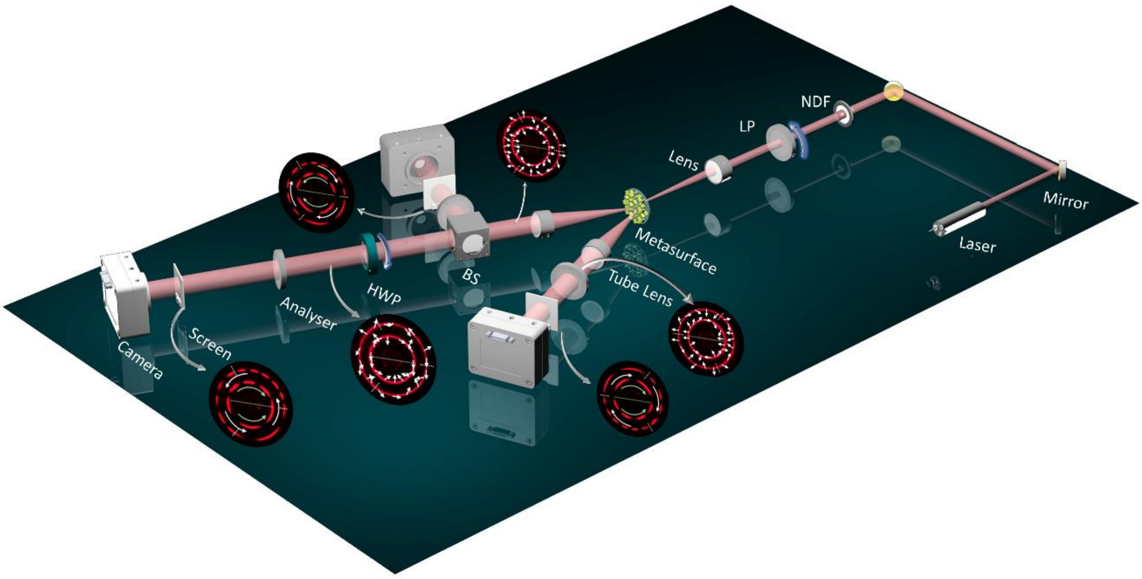

Fig. S6. Detailed schematic of the experimental setup. NDF: neutral density filter; LP: linear polarizer; BS: beam splitter; HWP: half-wave plate; Analyser: linear polarizer. The transmission axis of all the analysers is fixed along the vertical direction.

In the optical path, an LP is rotated (as indicated by an arrow) to control the polarization state of incident light. The lens ($f = 150\ \mathrm{mm}$) before metasurface is used to focus light onto metasurface and perform a Fourier transformation of generated beam. After light passes through the metasurface, two symmetrically distributed hybrid GPVVBs are generated along the horizontal direction (x-axis). Tube lenses in the path of both deflected beams are placed to acquire a collimated hybrid GPVVB. **Right Beam**: light after passing through an analyser is displayed on a screen and the resultant patterns are captured through a camera. **Left beam**: A beam splitter (BS) is used to split hybrid GPVVB into two beams. The original and modified hybrid GPVVB with a new polarization profile. The modulated intensity profile with unique features (e.g., lobes) of the original hybrid GPVVB can be obtained directly after passing through an analyser, while that of the modified hybrid GPVVB is obtained through the HWP and the analyser. A complete mechanism of polarization modification is explained in supplementary section 1 and Fig. 5 of the main text.

Supplementary Section 6: Kirchhoff diffraction integration

All the designed devices are simulated using the Kirchhoff diffraction integration. The actual size of the metasurfaces is used to calculate the far-field intensity profiles by the following equation [10, 11]:

$$E(x,y) = \frac{e^{ikz}}{i\lambda z} e^{\frac{ik}{2z}(x^2+y^2)} \iint E(x_0, y_0) e^{-\frac{ik}{z}(xx_o+yy_o)} dx_o dy_o\,, (S16)$$

where $E(x_0, y_0)$ is the complex amplitude profile of GPVVB at the $z = 0$. x_o and y_o are the coordinates of nanorods, while x and y are the coordinates of the observation plane at a distance of z. $k = \frac{2\pi}{\lambda}$ is the wavevector and λ denotes wavelength. In the simulation, the amount of time needed for the calculation depends on the resolution of the observation plane (x, y). In this work, the area of $200 \times 200\ \mu\mathrm{m}^2$ on the observation plane with the resolution of 0.3 µm is used in our simulation.

Supplementary Section 7: Metasurface Design

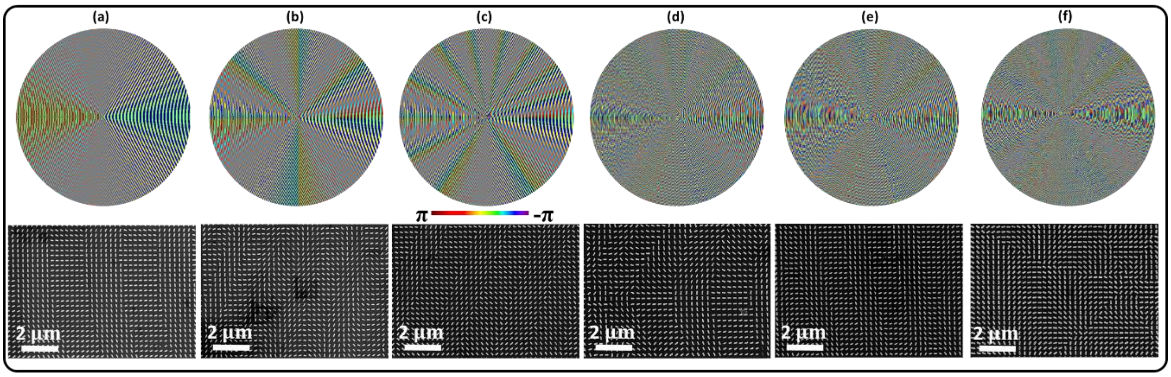

Fig. S7. Metasurface Design. 1st row shows phase profiles for GPVVBs with various polarization orders (a) $m_1, = +1, m_2 = -1$ (b) $m_1, = +4, m_2 = +2$ (c) $m_1 = +3, m_2 = +6, m_3 = +9$ and phase profiles for hybrid GPVVBs with polarization orders (d) Inner ring: $m_1 = -4, m_2 = -2$ and Outer ring: $m_1 = +3, m_2 = +6, m_3 = +9$. (e) Inner ring: $m_1 = -4, m_2 = +2$ and Outer ring: $m_1 = +3, m_2 = +6, m_3 = +9$. (f) First ring: $m_1 = -4, m_2 = -2$, second ring: $m_1 = +3, m_2 = +6, m_3 = +9$ third ring: $m_1 = -4, m_2 = -2$ and fourth ring: $m_1 = +3, m_2 = +6, m_3 = +9$. 2nd row shows the corresponding SEM images of the fabricated samples.

Supplementary Section 8: Higher-Order GPVVBs Generation and Manipulation

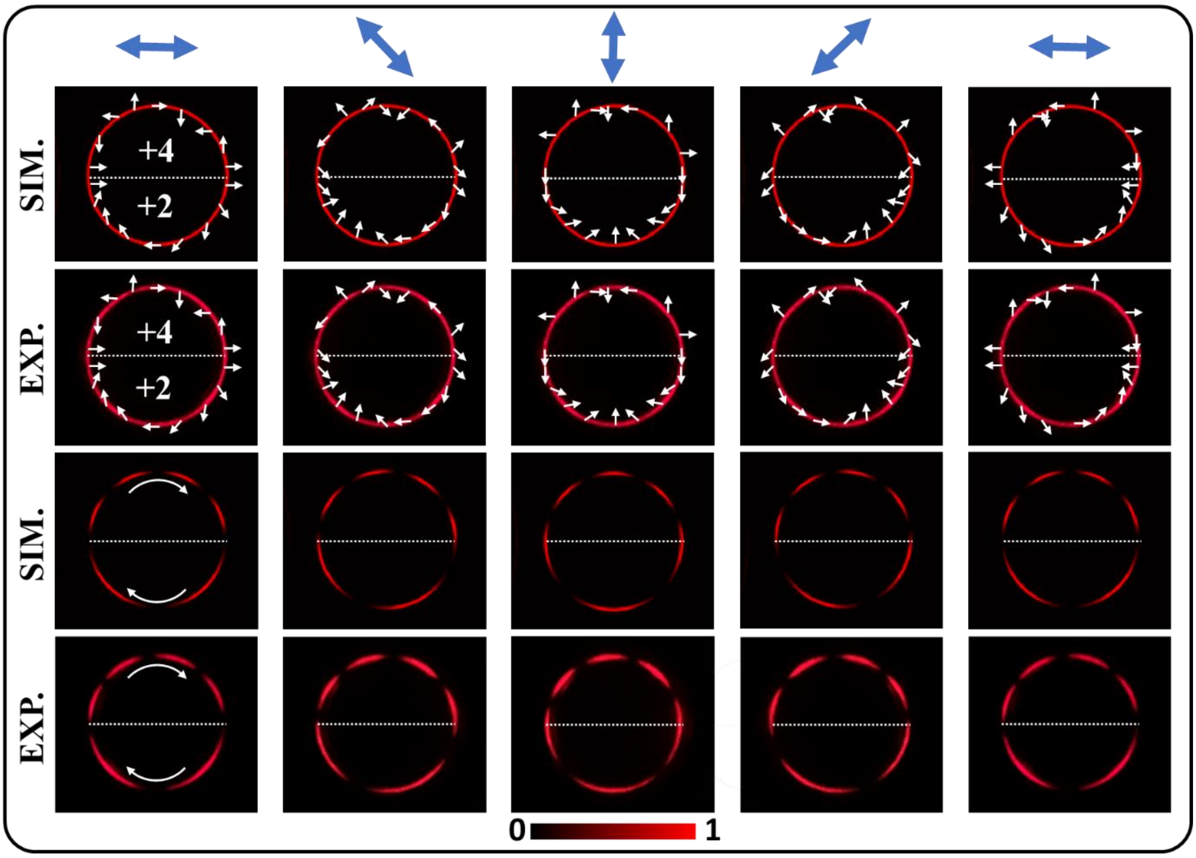

Fig. S8. Higher-Order GPVVB generation and manipulation by grafting two OVs. Simulated and measured intensity profiles for the polarization order $m_1 = +4$ (upper sector) and $m_2 = +2$ (lower sector). The white arrows depict various polarization profiles under the illumination of linearly polarized light with different polarization directions (blue arrows). Blue arrows on the top represent the direction of incident polarization. 1st and 2nd rows are intensity profiles without an analyser, which are modulated with an analyser (shown in 3rd and 4th rows). Curved arrows show the direction of rotation of the lobes.

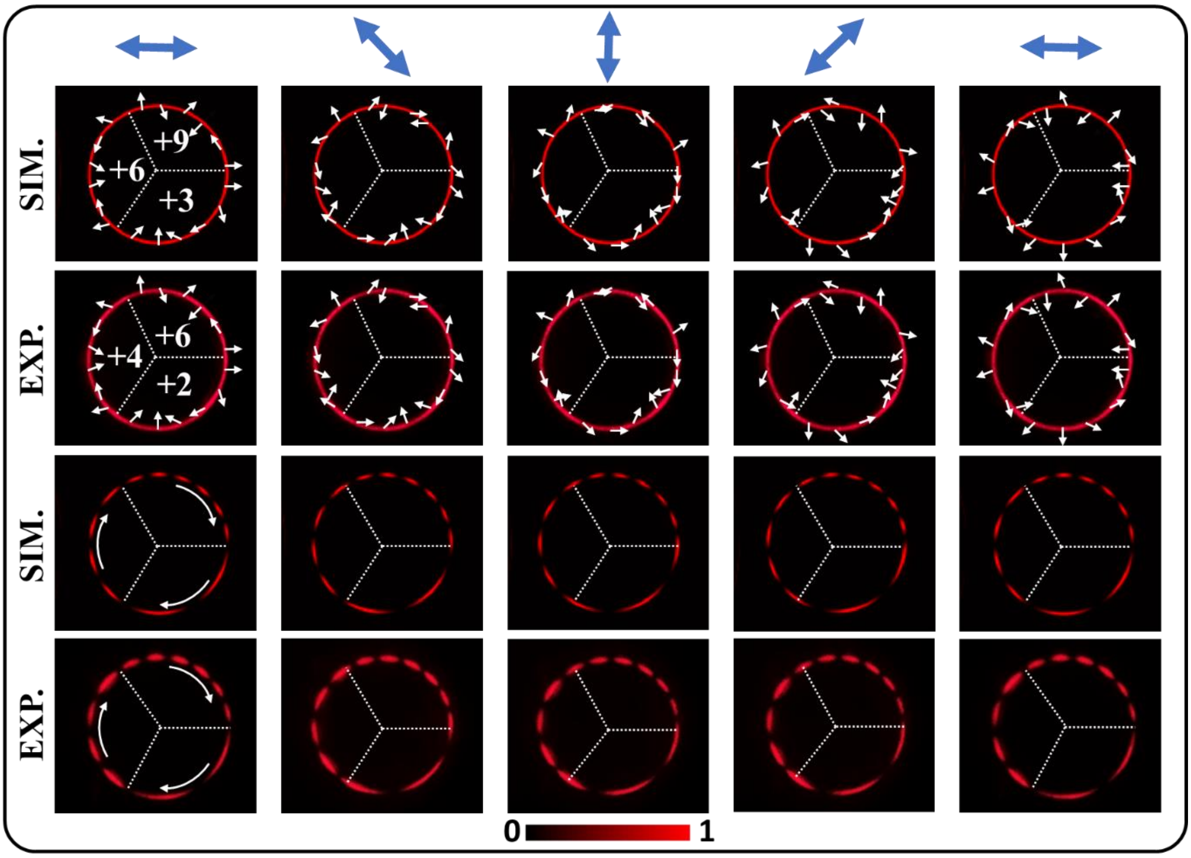

Fig. S9. Higher-Order GPVVB generation and manipulation by grafting three OVs. Simulated and measured intensity profiles for the polarization order $m_1 = +3, m_2 = +6$, and $m_3 = +9$. The white arrows depict various polarization profiles under the illumination of linearly polarized light with different polarization directions (blue arrows). Blue arrows on the top represent the direction of incident polarization. 1st and 2nd rows are intensity profiles without an analyser, which are modulated with an analyser (shown in 3rd and 4th rows). Curved arrows show the direction of rotation of the lobes.

Supplementary Section 9: Rotation Angle Details

Table S1: Rotation angles acquired for GPVVB with $N = 2$ (grafting of two OVs)

	θ_1			θ_2		
**Incident Polarization (degrees)**	**Theory**	**Simulation**	**Experiment**	**Theory**	**Simulation**	**Experiment**
0	0	0	0	0	0	0
45	11.25	14	13	22.5	24	22
90	22.5	23	23	45	44	44

135	33.75	35	34	67.5	69	69
180	45	44.5	41	90	87	88

Table S2: Rotation angles acquired for GPVVB with $N = 3$ (grafting of three OVs)

	$\boldsymbol{\theta_1}$			$\boldsymbol{\theta_2}$			$\boldsymbol{\theta_3}$		
**Incident Polarization (degrees)**	**Theory**	**Simulation**	**Experiment**	**Theory**	**Simulation**	**Experiment**	**Theory**	**Simulation**	**Experiment**
0	0	0	0	0	0	0	0	0	0
45	15	15	14	7.5	12	12	5	3	4
90	30	30	35	15	17	17.5	10	8	10
135	45	43	43	22.5	23	21	15	15	13
180	60	62	60	30	34	29	20	18	17.5

Supplementary Section 10: Polarization Measurement

The polarization state of the output light beam is obtained using the Stokes polarimetry [1, 12]. By measuring the series of intensity profiles by adding an analyser, the stokes parameters can be calculated. The Stokes parameters can be expressed as [13]:

$$S_o = I(0^o) + I(90^o), (S17)$$

$$S_1 = I(0^o) - I(90^o), (S18)$$

$$S_2 = I(45^o) - I(135^o), (S19)$$

Here $I(0^o)$, $I(45^o)$, $I(90^o)$, $I(135^o)$ are the intensities of GPVVBs after an analyser rotated at $0^o, 45^o, 90^o, 135^o$ with respect to the $x\ axis$. The spatial distribution of polarization states as shown in Fig. 4a can be calculated as [13]:

$$\vartheta = \frac{1}{2}\arctan\left(\frac{s2}{s1}\right), (S20)$$

Supplementary Section 11: Hybrid GPVVB Generation and Manipulation

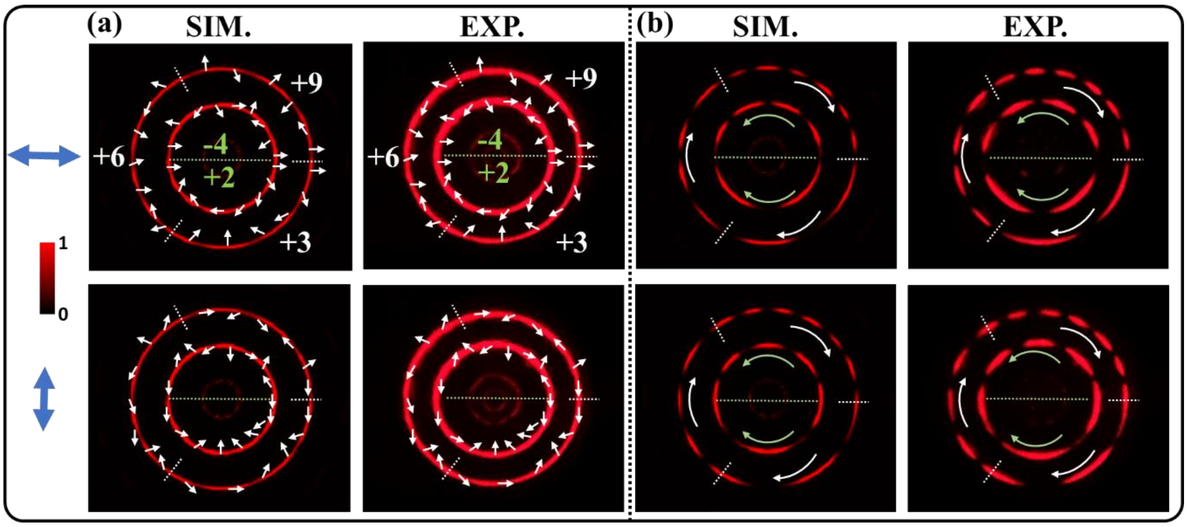

Fig. S10. Hybrid GPVVB Generation. (a) Intensity profile and polarization distribution of a hybrid GPVVB under the illumination of linearly polarized light along horizontal and vertical directions. Blue double arrows and white arrows show the linear polarization direction of incident light and that of the hybrid GPVVB, respectively. **(b)** Modulated intensity profiles of the hybrid GPVVB after passing through an analyser. The rotation of lobes in different directions indicates the existence of various polarization orders. Rotation directions of the inner and outer rings are shown in green and white curved arrows, respectively.

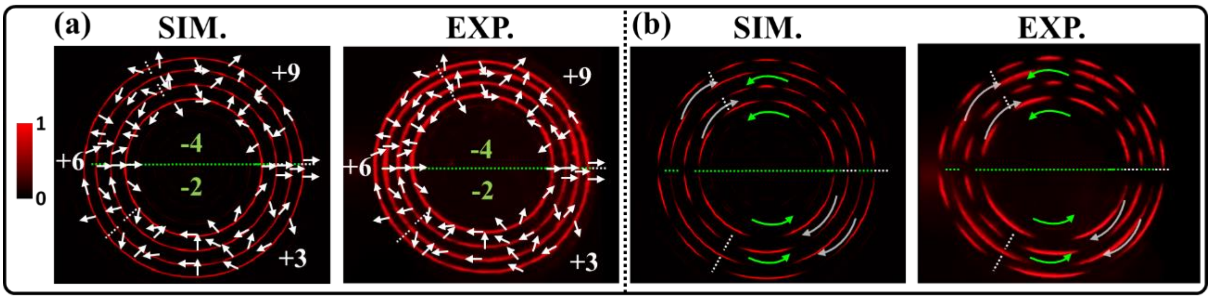

Fig. S11. Hybrid GPVVB Generation with four higher order GPVVBs. (a) Intensity profile and polarization distribution of a hybrid GPVVB under the illumination of linearly polarized light along the horizontal direction. Blue double arrows and white arrows show the linear polarization direction of incident light and that of the hybrid GPVVB, respectively. (b) Modulated intensity profiles after passing through an analyser. The rotation of lobes in different directions indicates the existence of various polarization orders. The rotation directions of the rings are shown in green and white curved arrows, respectively. The first and

third ring is formed by grafting two OVs while the second and fourth ring is formed by grafting three OVs. Here the axicon periods for four rings are $u_1 = 4\ \mu\text{m}$, $u_2 = 3.3\ \mu\text{m}, u_3 = 2.8\ \mu\text{m}\ and\ u_4 = 2.5\ \mu\text{m}$, respectively.

Supplementary Section 12: Lobe Size Modulation

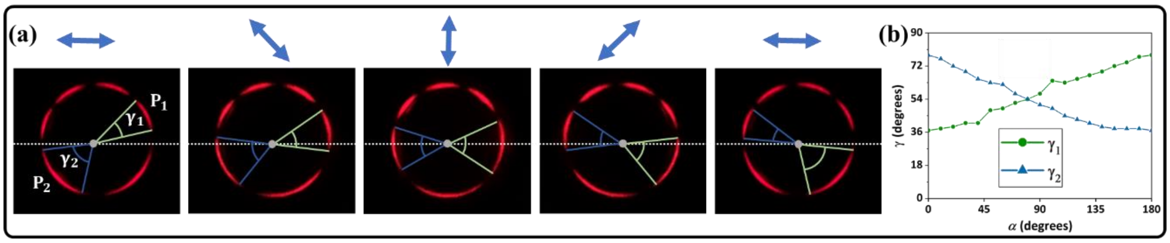

Fig. S12. Effect of incident polarization (α) on the size of the lobe. (a) Two lobes, indicated by P_1, and P_2, are selected to study the change. The initial size of each corresponding lobe is denoted by the arc angle γ_1 and γ_2. As the α increases from $0°$ to $180°$, P_1 and P_2 start to rotate in opposite sectors, which causes an increase in γ_1 and a decrease in γ_2. **(b)** Relationship between arc angles and α. A linear relationship can be seen, which can be analytically given as $\gamma_1 = 32.404 + 2.4702\alpha$ and $\gamma_2 = 78 - 2.4579\alpha$. The corresponding correlation coefficients are 0.987 and 0.966, respectively.

References

1. Bao Y, Ni J, Qiu CW. A minimalist single-layer metasurface for arbitrary and full control of vector vortex beams. *Adv. Mater.* 2020, **32**(6)**:** 1905659.

2. Vaity P, Rusch L. Perfect vortex beam: Fourier transformation of a Bessel beam. *Opt. lett.* 2015, **40**(4)**:** 597-600.

3. Maurer C, Jesacher A, Fürhapter S, Bernet S, Ritsch-Marte M. Tailoring of arbitrary optical vector beams. *NJPh* 2007, **9**(3)**:** 78.

4. Li D, Chang C, Nie S, Feng S, Ma J, Yuan C. Generation of elliptic perfect optical vortex and elliptic perfect vector beam by modulating the dynamic and geometric phase. *Appl. Phys. Lett.* 2018, **113**(12)**:** 121101.

5. Li P, Zhang Y, Liu S, Ma C, Han L, Cheng H*, et al.* Generation of perfect vectorial vortex beams. *Opt. lett.* 2016, **41**(10)**:** 2205-2208.

6. Yue F, Wen D, Zhang C, Gerardot BD, Wang W, Zhang S*, et al.* Multichannel polarization-controllable superpositions of orbital angular momentum states. *Adv. Mater.* 2017, **29**(15)**:** 1603838.

7. Zhou Z, Li J, Su R, Yao B, Fang H, Li K*, et al.* Efficient silicon metasurfaces for visible light. *Acs Photonics* 2017, **4**(3)**:** 544-551.

8. Lin D, Fan P, Hasman E, Brongersma ML. Dielectric gradient metasurface optical elements. *Science* 2014, **345**(6194)**:** 298-302.

9. Deng Z-L, Deng J, Zhuang X, Wang S, Li K, Wang Y*, et al.* Diatomic metasurface for vectorial holography. *Nano. Lett.* 2018, **18**(5)**:** 2885-2892.

10. Intaravanne Y, Wang R, Ahmed H, Ming Y, Zheng Y, Zhou Z-K*, et al.* Color-selective three-dimensional polarization structures. *Light Sci. & Appl.* 2022, **11**(1)**:** 302.

11. Zhang Y, Liu W, Gao J, Yang X. Generating focused 3D perfect vortex beams by plasmonic metasurfaces. *Adv. Opt. Mater.* 2018, **6**(4)**:** 1701228.

12. Song Q, Baroni A, Wu PC, Chenot S, Brandli V, Vézian S*, et al.* Broadband decoupling of intensity and polarization with vectorial Fourier metasurfaces. *Nat. Commun.* 2021, **12**(1)**:** 3631.

13. Liu M, Huo P, Zhu W, Zhang C, Zhang S, Song M*, et al.* Broadband generation of perfect Poincaré beams via dielectric spin-multiplexed metasurface. *Nat. Commun.* 2021, **12**(1)**:** 2230.
