# Supplementary material for: Dynamic control of hybrid grafted perfect vector vortex beams
Source: Nat Commun. 2023 Jul 3;14:3915. doi: 10.1038/s41467-023-39599-8 (PMC10318044; doi:10.1038/s41467-023-39599-8)
Supplement: Supplementary file 3 — Description of additional supplementary files [file 41467_2023_39599_MOESM3_ESM.pdf]

<u>**Description of additional supplementary files**</u>

**Supplementary Movie 1:** Polarization and intensity evolution for GPVVB with polarization order $\mathrm{m}_1 = -1$ and $\mathrm{m}_2 = +1$.

**Supplementary Movie 2**: Polarization and intensity evolution for GPVVB with polarization order $\mathrm{m}_1 = +2$ and $\mathrm{m}_2 = +4$.

**Supplementary Movie 3**: Description: Polarization and intensity evolution for GPVVB with polarization order $\mathrm{m}_1 = +3$, $\mathrm{m}_2 = +6$ and $\mathrm{m}_3 = +9$.

**Supplementary Movie 4**: Description: Evolution of rotation angle of lobes for various sectors of GPVVBs.

**Supplementary Movie 5**: Polarization and intensity evolution for Hybrid GPVVB with polarization order $\mathrm{m}_1 = -2$, and $\mathrm{m}_2 = -4$ (inner ring) and $\mathrm{m}_1 = +3$, $\mathrm{m}_2 = +6$ and $\mathrm{m}_3 = +9$ (outer ring).
